# Utilizing 7 Tesla QSM to evaluate the relationship between cerebral small veins and microbleeds in Alzheimer’s Disease

**DOI:** 10.1002/alz.089888

**Published:** 2025-01-09

**Authors:** Laya Ashouri

**Affiliations:** ^1^ Urmia University of Medical Science, Urmia, West Azarbayjan Iran (Islamic Republic of)

## Abstract

**Background:**

Cerebral microbleeds (CMBs) are small hypointense round lesions that indicate leakage of blood products from cerebral vessels damaged by β‐amyloid‐40 (Aβ) and typically are detected by T2*‐weighted and susceptibility weighted imaging (SWI) on MRI. They are indicators of cerebral small vessel diseases, especially cerebral amyloid angiopathy (CAA), affecting cortical small arteries. Quantitative susceptibility mapping (QSM) is an advanced MRI imaging technique used to quantify the magnetic susceptibility of tissues in the human body. In this study, we utilized the QSM technique to analyze venous structures at ultra‐high resolution in a cohort of patients with AD and age‐matched control subjects using 7T MRI. Our aim is to evaluate the feasibility of using ultra‐high resolution QSM at 7T MRI to find a link between small veins and CMBs in individuals with AD.

**Method:**

In‐vivo human imaging was performed using a 7T MRI scanner and were analyzed by two neuroradiologists. CMBs were defined as hyperintense, round lesions in the QSM images. For verification, their appearance was matched on the SWI images, where CMBs are hypointense and associated with. CMBs with an immediate adjacent to a vein were assessed in the QSM images. They were defined as CMBs with small veins connection.

**Result:**

This high‐resolution 7T neuroimaging study investigated whether a spatial relationship between small veins and CMBs could be detected in a cohort of AD patients. QSM enabled depiction of cerebral CMBs with a direct connection to a vein and their localization. Our data support the notion that CMBs relate anatomically to veins. Understanding the role of vein pathology in CAA and its role in neurodegenerative pathologies might open novel insights in the pathophysiology of AD.

**Conclusion:**

A key strength of our study lies in the utilization of high‐resolution QSM at 7T MRI. High‐resolution MRI enhances the visibility of small structures, and when maintained at the same resolution, imaging at a higher field MRI increases the signal‐to‐noise ratio. QSM elevates CMB detection sensitivity while facilitating precise visualization of the brain's venous vasculature. Notably, the postprocessing algorithm ensures a high level of specificity for venous blood susceptibilities, minimizing the likelihood of measuring arterioles or slow‐flow small arteries.

